# Notes of a protein crystallographer: the legacy of J.-B. J. Fourier – crystallography, time and beyond

**DOI:** 10.1107/S205979832100293X

**Published:** 2021-04-26

**Authors:** Celerino Abad-Zapatero

**Affiliations:** a Institute of Tuberculosis Research, Center for Biomolecular Sciences, Department of Pharmacological Sciences, University of Illinois at Chicago, Chicago, IL 60607, USA

**Keywords:** J.-B. J. Fourier, heat transmission, concept of time, Bergson–Einstein debate, nonequilibrium thermodynamics

## Abstract

The importance of the Fourier transform as a fundamental tool for crystallo­graphy is well known in the field. However, the complete legacy of Jean-Baptiste Joseph Fourier (1768–1830) as a pioneer Egyptologist and premier mathematician and physicist of his time, and the implications of his work in other scientific fields, is less well known. Significantly, his theoretical and experimental work on phenomena related to the transmission of heat founded the mathematical study of irreversible phenomena and introduced the flow of time in physico-chemical processes and geology, with its implications for biological evolution. Fourier’s insights are discussed in contrast to the prevalent notion of reversible dynamic time in the early 20th century, which was dominated by Albert Einstein’s (1875–1953) theory of general relativity versus the philosophical notion of *durée* proposed by the French philosopher Henri-Louis Bergson (1859–1941). The current status of the mathematical description of irreversible processes by Ilya Romanovich Prigogine (1917–2003) is briefly discussed as part of the enduring legacy of the pioneering work of J.-B. J. Fourier, first established nearly two centuries ago, in numerous scientific endeavors.

## Abstract 2

The legacy of J.-B. J. Fourier, a pioneer Egyptologist and premier mathematician and physicist of his time, in crystallography and many other scientific fields is reviewed in the context of nearly two centuries having passed since his landmark publication on *The Analytical Theory of Heat* (1822). The legacy of Fourier’s work in crystallography and many other scientific fields is reviewed with an emphasis on current developments in the study of nonequilibrium thermodynamics.

## 1. Introduction   

Nearly two centuries ago, in 1822, Jean-Baptiste Joseph Fourier published his milestone *Théorie analytique de la chaleur*, published much later translated into English as *The Analytical Theory of Heat* (Fourier, 1878 ▸). In an earlier essay, submitted to the Royale Académie des Sciences as an entry for the 1811 prize competition on the subject of the propagation of heat in solid bodies (published by the Royal Academy in 1826; Fourier, 1826 ▸), Fourier presented the notion that it is possible to express any ‘irregular’ function (including discontinuous functions) as a sum of ‘waves’ represented by a sum of sine and cosine functions. The application of this mathematical concept to the periodic distribution of X-ray scatterers in a crystalline array hypothesized by W. H. Bragg (1862–1942) (Bragg, 1915 ▸) developed into the foundations of crystallography as we know it today. The application of ‘Fourier methods’ to solve crystal structures has been covered by Isaacs (2016 ▸) in the context of the history of experimental phasing methods in macromolecular crystallography. A more detailed discussion will be presented here, emphasizing the development of the concepts and relevant crystal structures, as well as the often-overlooked importance of the improvements in effective, fast and storage-efficient computational algorithms for calculation of the discrete Fourier transform (DFT).

Aside from the implications and importance of Fourier’s work in crystallography and mathematics (*i.e.* boundary problems and rigorous definition of functions, among others) and physics, the equation that Fourier unveiled to the world in 1822 described mathematically the heat flow on a solid object, thus initiating the study of irreversible phenomena in nature. From this early memoir about the propagation of heat in solids, submitted to the Institute de France (Fourier, 1807 ▸), and through years of intense effort while in Grenoble, Fourier expanded his insights into heat transmission and loss to describe mathematically (i) the communication of heat between discrete bodies, (ii) terrestrial heat and temperature distributions on earth and (iii) radiant heat and the movement of heat in fluids. Although mainly recognized for his theor­etical work, Fourier also conducted experiments measuring transient and steady-state temperature distributions to devise the parameters of thermal conductivity in a heated annulus and the cooling rate in a sphere (Fourier, 1826 ▸; discussed in detail in Narasimhan, 2010 ▸).

The equation of heat flow inspired other physicists to describe other physical phenomena even in Fourier’s lifetime. Georg Simon Ohm (1789–1854) acknowledged the inspiration of Fourier’s equation to formulate the well known equation for the flow of electricity (Ohm, 1827 ▸), and further applications in the mathematical analysis of electricity and magnetism followed (Green, 1828 ▸). Later, William Thompson (Lord Kelvin; 1824–1907) published work directly relating the motion of heat in solids to the mathematical theory of electricity and its applications (Thompson, 1842 ▸).

Surprisingly, Fourier’s work related to terrestrial heat would have an unexpected application in geology, with unforeseen consequences for biology. During his stay in France, early knowledge of Fourier’s equations for the transmission of heat and his interest in geology led Lord Kelvin to early estimates of the age of the Earth. In two separate communications to the leading geological societies of the time (Thompson, 1864 ▸, 1871 ▸), he estimated the age of the Earth to be between 20 and 400 (probably 100) million years, originating the notion of geological time in the evolution of the Earth’s crust and the ensuing biological evolution. These limits for the age of the Earth were considered as a serious objection to Darwin’s evolution by natural selection, which admittedly required longer time periods (Darwin, 1872 ▸). Later, more adequate dating methods based on radioactive decay eventually yielded a more extended age for the Earth (∼4.5 billion years), which was more in agreement with the expectations of Lord Kelvin that anticipated all sciences to be consistent with the tenets of the more advanced physical sciences. See Halls & Wilkie (2015 ▸) for a more detailed discussion of the controversy and the influence of Lord Kelvin in estimating the age of the Earth.

The cycle of continuing years brings the notion of time more into the human scale of events. Yet, scientific luminaries such as Albert Einstein have often expressed the notion that time, and more precisely the distinction between past and present, was an ‘illusion’ (Isaacson, 2007 ▸). Scientists and philosophers have debated these two conflicting views of human experience innumerable times. A comprehensive but accessible review of the ideas related to this clash was published a few years ago by the science historian Professor J. Canales (Canales, 2015 ▸), focusing on the historical debate between Einstein and the most prominent French philosopher of the time, Henri-Louis Bergson, in Paris on 6 April 1922 at the French Philosophical Society. Surprisingly, this significant event for the history of Western thought was not discussed or even mentioned in a comprehensive and highly praised biography of Einstein (Isaacson, 2007 ▸). The physicist was at the peak of his fame after the confirmation of the general theory of relativity, while Bergson published two very influential philosophical oeuvres in which the notion of time had a primordial importance: *L’Évolution Crèatrice* (1906) and *Durée et Simultanéité* later in 1922. Bergson, a professor of the prestigious École Normale de France, presided over French philosophy for many years and went on to receive the Nobel Prize in Literature in 1927 for the former book.

The influence of the Bergson–Einstein debate in the years that followed was such that philosophical schools of thought could essentially be distinguished by whether they aligned with one or the other in their respective conceptions of time. For instance, Alfred North Whitehead (1861–1947), a premier British mathematician and philosopher who later moved to Harvard, was the mentor and thesis advisor of no other than Bertrand Arthur William Russell (1872–1970), and together they co-authored the three-volume *Principia Mathematica* (1910), a milestone in Western philosophy and mathematical logic. Yet, they departed in their respective philosophies: Whitehead was a strong advocate of Bergson’s concepts, while Russell, who had acrimonious debates and correspondence with Bergson, was unable to accept his views.

In addition to myriad applications of Fourier’s insights, the ensuing advances in the study of nonequilibrium phenomena in physics, chemistry and biology, particularly during the 20th century, should make us re-evaluate the significance of Fourier’s early contributions to the study of heat transmission nearly two centuries ago. Furthermore, the implications of his work, and the revised conceptions of ‘time’ that ensue, are well beyond the conflicting views of the Bergson–Einstein debate. May this be our homage to the legacy of J.-B. J. Fourier, which extends well beyond his critical role in our field of study and the myriad other scientific endeavors in which the Fourier transform is used in its different formulations.

## 2. Biographical background   

J.-B. Joseph Fourier was born in the French town of Auxerre on 21 March 1768. The town is located one hundred miles southeast of Paris, and by 1751 was a prosperous town with a large ecclesiastical community centered around the Abbey of Saint-Germain on the banks of the river Yonne, a tributary of the Seine. All of these factors were probably significant when Fourier’s father, a master tailor, settled the family in Auxerre. Fourier was the ninth of 12 children from his father’s second marriage, but was left an orphan a little before his tenth birthday. This dramatic turn of events does not seem to have affected his future accomplishments.

**Figure 1:**
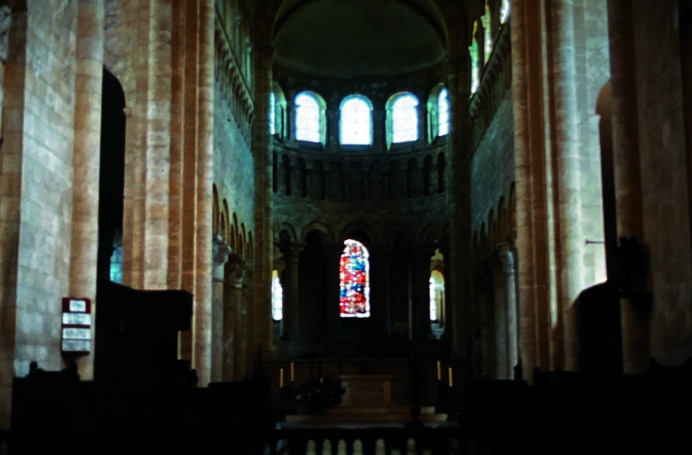
View of the interior of the magnificent Romanesque abbey of Saint-Benoît-sur-Loire, where Fourier first received his mathematical education, later becoming a teacher himself. (Author’s personal collection.)

As was commonplace in those days, his education was connected to the church, first at Saint-Germain and later at the magnificent Romanesque abbey of Saint-Benoît-sur-Loire, Burgundy, a military school run by Benedictine monks (Fig. 1 ▸). He showed precocious ability in mathematics, and after completing his education he served as a teacher to the following generations of pupils entering the school.

The ideals and vortices of the French Revolution also reached the provincial government of Auxerre and, while teaching, he was also a member of the revolutionary committee, later becoming president. He was subsequently arrested and freed twice in Auxerre. An unexpected nomination to be a pupil at the École Normale, which had recently been founded for the purpose of educating elementary-school teachers, allowed him to escape, at least temporarily, the turmoil in Auxerre and go to Paris as a ‘Normalien’.

Fourier turned out to be the most capable, and later the most illustrious, of the early generations of students. His political activities back in Auxerre caught up with him and he was arrested again, with one of the charges being having inspired terrorism. The reasons for Fourier’s final release are not known. However, it is almost certain that the intervention of Pierre-Simon Laplace (1749–1827), Joseph-Louis Lagrange (1736–1813) and Gaspar Monge (1746–1818), who had already recognized Fourier’s mathematical talents, was crucial. More important at the time was his recognized ability as a teacher of mathematics. Thus, fortunately for science in general and for crystallography in particular, he did not follow the tragic fortune of his contemporary Antoine-Laurent Lavoisier (1743–1794).

The revolutionary years ended with the political coup of Napoleon Bonaparte (1769–1821), and Fourier resurfaced in Paris as a professor and administrator at the renamed École Polytechnique, where among others he mentored a brilliant student, namely Siméon Denis Poisson (1781–1840; of Poisson’s distribution and many other contributions bearing his name). The new political turn of events had a tremendous impact on Fourier’s life. In 1798, a select group of intellectuals, scientists, artists, engineers and a military force of 30 000 soldiers and sailors sailed from Toulon to a secret destination led by Napoleon himself; Fourier was included as a senior member and professor of the École Polytechnique. Admiral Nelson’s squadron scoured the Mediterranean looking for the French armada, and came within two miles but without any military action. Soon the French soldiers moved towards Cairo, and on 24 July Bonaparte entered the capital. However, the French fleet was essentially destroyed a few days later in Aboukir Bay, and the prospects of returning home looked grim. Among the chaos that ensued, Napoleon managed to create by decree the Cairo Institute, including as members the scientific and intellectual elite of France: Monge, Fourier (mathematicians), Claude Louis Berthollet (1748–1822; chemist), Nicolas-Jacques Conté (1755–1805; engineer and inventor) and Etienne Geoffroy Saint-Hilaire (1772–1844; naturalist), among others. Having observed the administrative skills of Fourier during the journey, Bonaparte appointed him permanent secretary of the Institute. Sessions of the members were scheduled to take place regularly in the large harem room of the Palace of Beis (Herivel, 1975 ▸).

Bonaparte’s agenda for the institute included many practical questions (such as what is more practical in Cairo: windmills or watermills?), but the members of the Institute, driven by their own curiosity and interests, assembled and collected an enormous amount of material and artifacts on many different aspects of Egyptian natural history, art, monuments and culture. This was later edited and published in a multivolume collection entitled *Description of Egypt* super­vised and prefaced by Fourier himself between 1808 and 1825.

**Figure 2:**
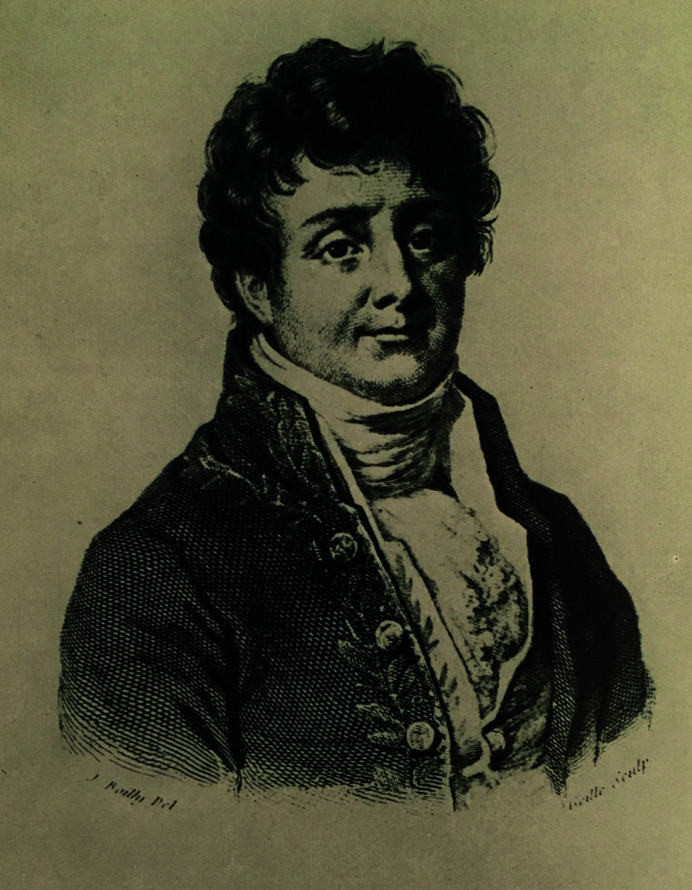
Jean-Baptiste Joseph Fourier: lithograph by Louis Boilly (1823). From a copy in the possession of the Archives of the Académie des Sciences, Paris. Reproduced with permission, courtesy of the Academy of Sciences, Archives and historical heritage.

Upon the return of what was left of the Egypt expedition to France, Napoleon recognized Fourier’s scientific and administrative contributions as the permanent secretary of the Cairo Institute and appointed him Prefect of the Department of Isère (capital Grenoble). There he began his systematic quest towards understanding the transmission of heat in solids (Fourier, 1807 ▸), which continued during the years 1802–1814. Historians have argued that his long visit to warmer climates made it difficult for him to adapt to the cold and damp climate of Grenoble, and he was particularly sensitive to cold; he had developed chronic rheumatic pains. Most probably, this prompted his interest in heat transmission and heat loss in solids. Besides his contributions to science and completing his Egyptological work, as Prefect of Isère Fourier demonstrated extraordinary administrative ability, directing the drainage of the swamps of Bourgoin covering over twenty million acres, which were the cause of annual fever epidemics and high mortality rates in the area. In 1809 Napoleon made him a baron (Herivel, 1975 ▸; Fig. 2 ▸).

**Figure 3:**
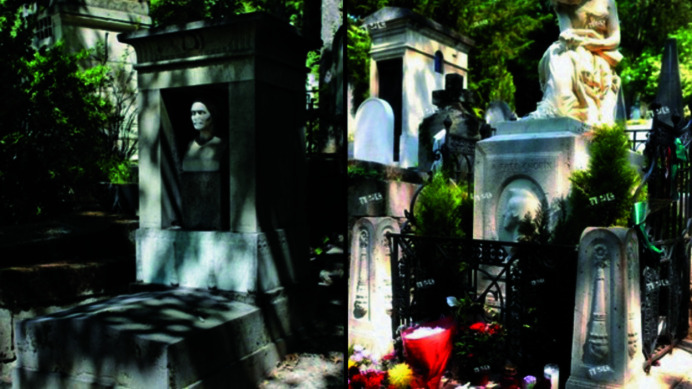
Left: the tomb of J.-B. J. Fourier in the Cimetière du Père Lachaise in Paris. The figure and style relate to his research during the Napoleonic expedition to Egypt and his position as permanent secretary of the Cairo Institute. Right: the tomb of Frédéric Chopin in the same cemetery. (Author’s personal collection obtained from a public domain source: commons.wikimedia.org.)

After the fall of Napoleon in 1815, Fourier returned to Paris and was appointed director of the Statistical Bureau of the Seine. This appointment allowed him to devote more time to his work on the theory of heat transmission and loss. Elected to the Académie des Sciences in 1817, he became perpetual secretary in 1822. The merit of his work in Egyptology was also recognized by his election as a member of the French Académie des Sciences, and of the Académie National de Médecine in 1826. As permanent secretary of the Académie he was at the center of scientific activity in France and saw the publication of his memoir on heat transmission (Fourier, 1826 ▸) receiving many scientific honors, among them election to the Royal Society of London. His deteriorating health led to a fatal heart attack on 16 May 1830. He was buried in the Cimetière du Père Lachaise in Paris, the place of burial of many illustrious names, Frédéric Chopin (1810–1849) among them. A suitable memorial related to his achievements in Egyptology was erected at his grave (Herivel, 1975 ▸; Fig. 3 ▸), financed by friends and colleagues including the noted mathe­matician Sophie Germain (1776–1831) and the famous naturalist Georges Cuvier (1769–1832).

## 3. The development of Fourier methods for the solution of crystal structures   

Reviewing the growing field of crystallography at the Fifth International Congress of the IUCr in 1960 in Cambridge, UK (published in Bragg, 1962 ▸), the younger Bragg (William Lawrence Bragg, 1890–1971) summarized the achievements of the initial period of the field (1912–1920) with six highlights: (i) the establishment of the wavelength of X-rays, (ii) the solution of several simple crystal structures with only one unknown parameter that could be fixed by symmetry, (iii) the accurate measurement of the diffracted intensity of the reflections using his father’s X-ray spectrometer, (iv) the determination and confirmation by measurement of the Debye correction term due to atomic motion, (v) C. G. Darwin’s formulae for the reflection intensity of perfect and mosaic crystals and (vi) the realization and suggestion by his father that crystal diffraction can be related to the Fourier components of the scattering elements (atoms) of the crystal.

The last insight was presented by the elder Bragg (William Henry Bragg, 1862–1942) at the Bakerian Lecture on 18 March 1915 and was later published in the *Proceedings of the Royal Society* (Bragg, 1915 ▸). He introduced the notion of a periodic variation of scatterers in the crystal by writing

Let us imagine then that the periodic variation of density has been analyzed into a series of harmonic terms. The coefficient of any term will be proportional to the intensity of the reflection to which it corresponds.

(Bragg, 1915 ▸). Assuming a periodic distribution for the atomic scatterers, he ends the preliminary analysis with the following Fourier series for a one-dimensional density function,
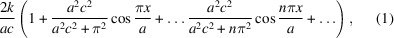
where *a* = *d*/2 is the spacing of the atoms in one dimension of the crystal, *d* is the plane spacing and *c* is related to an exponential function describing the density of the atoms.

However, after this initial suggestion, it took another 15 years and several publications from the laboratory of William Duane (1872–1935) in Harvard and his postdoctoral student Robert James Havighurst (1900–1991) to deduce the modern expression for the electron density as a Fourier sum with the structure factors as coefficients. Duane also recognized the problem of phasing the structure factors which, in the simplest case of NaCl, he showed could be solved by symmetry arguments (Duane, 1925 ▸). Duane and Havighurst used one-dimensional Fourier series to determine the distribution of scattering matter in NaCl and various crystals along cell edges and face diagonals (Havighurst, 1925*a* ▸,*b* ▸).

The solution of several mineral structures by the measurement of absolute intensities with Bragg’s X-ray spectrometer, referred to the integrated (400) reflection of rock salt (Mo *K*α) as a standard, was critical to put the Fourier equations on an absolute scale in relation to the atomic numbers of the components of the crystals (atomic scattering factors). The first was barytes (BaSO_4_; James & Wood, 1925 ▸), with 11 parameters. The structure was confirmed by comparing calculated and observed structure factors. From this point on, the publications of new structures began to include numerical comparisons of the amplitudes ‘instead of the mystic letters v.s., s, m, w, v.w. for very strong to very weak’ (Bragg, 1962 ▸).

The application of this quantitative approach to the measurement of the absolute reflection intensities allowed the unraveling of the structures of silicates, which had been a goal for chemists and mineralogists alike. The breakthrough was the solution of diopside [MgCa(SiO_3_)_2_] by Warren & Bragg (1928 ▸), requiring the determination of 14 parameters. Although the tetrahedral symmetry of the SiO_4_ groups had been recognized before, the structure revealed tetrahedral groups linked through the corners to give the overall SiO_3_ stoichiometry.

**Figure 4:**
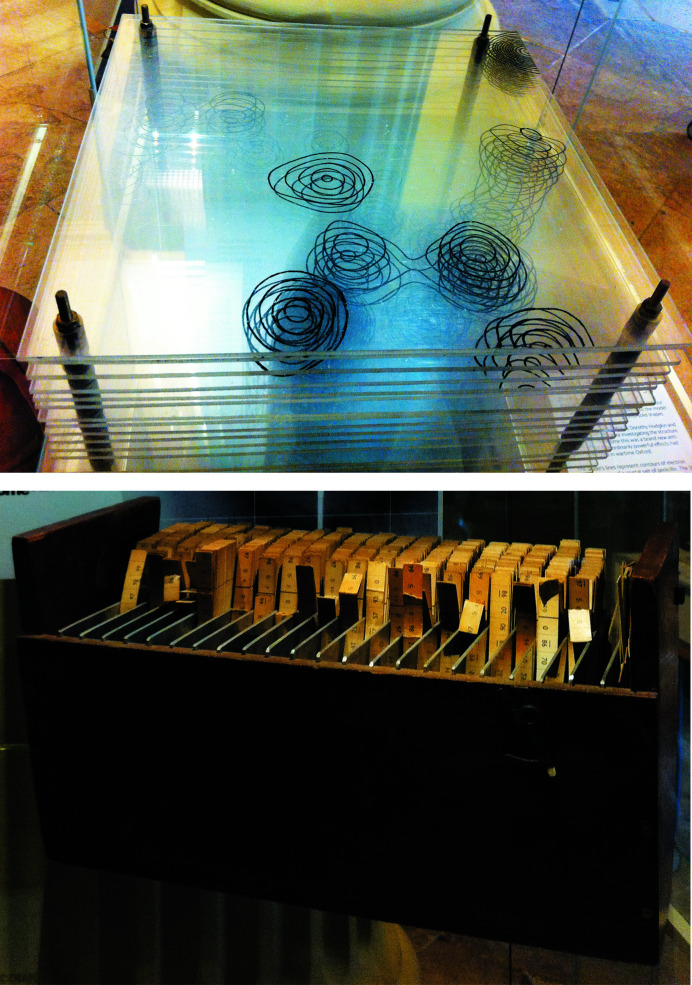
The early tools of crystallography. Top: one of the first three-dimensional electron-density maps, assembled from two-dimensional transparent sections spaced appropriately, used to determine the unknown chemical structure of penicillin by D. Hodgkin and collaborators in the mid-1940s. Bottom: the ubiquitous Beevers–Lipson strips, the tool of choice to calculate Fourier transforms prior to the advent of electronic computers. The image shows the set owned by D. Hodgkin. From the Oxford Museum of Science. (Author’s personal collection.)

The significance of this structure for the rigorous use of the Fourier transform in crystallography is that it served one year later to demonstrate the use of two-dimensional Fourier analysis to determine the positions of the atoms. W. L. Bragg spent four months as a visiting professor at the Massachusetts Institute of Technology and had brought with him an excellent and complete set of quantitative intensity measurements from crystals of diopside to a maximum value of sinθ for the three principal zones of the crystal. Calculation of the three two-dimensional Fourier projections of diopside revealed peaks corresponding to calcium, magnesium, silicon and oxygen. The atomic positions derived from solutions of the three different projections of the structure were in agreement (within 0.5%) with those published in the original structure a year earlier (Warren & Bragg, 1928 ▸). Although the elder Bragg had some significant insights into the handling of the data, this analysis was published by the younger Bragg in a landmark publication with the revealing title *The determination of parameters in crystal structures by means of Fourier series* (Bragg, 1929 ▸). In this publication, Bragg introduced the now standard nomenclature of |*F*_*hkl*_| for the amplitudes and (*F*_*hkl*_) for the Fourier coefficients, for which the signs (phases) need to be determined. The study of ‘organic crystals’ (as opposed to mineral crystals) and the effective and extended use of Fourier methods, including ‘Patterson methods’ (Patterson, 1934 ▸), combined with the presence of heavier atoms in the crystals, allowed the successes with penicillin (Crowfoot *et al.*, 1949 ▸; Figs. 4 ▸ *a* and 4 ▸ *b*) and vitamin B_12_ (Hodgkin *et al.*, 1956 ▸), the latter being ‘in a class of its own’ (C_63_H_89_CoN_14_O_14_P; Brink *et al.*, 1954 ▸; Hodgkin *et al.*, 1956 ▸; Bragg, 1962 ▸). These landmark structures established beyond question the value of crystallography in deciphering the three-dimensional structures of unknown chemical entities, including the protein structures that would soon follow.

The importance of Fourier series and the Fourier transform in crystallography, as formulated today, has been profusely presented in historical reviews and textbooks; it will not be reviewed here. It is indeed the basis of the field from phase determination to structure solution to structure refinement. This is also true for the extension of structure determination to larger macromolecular aggregates using cryo-EM and also MicroED.

## 4. The calculation of the Fourier transform in crystallography   

The historical 1929 Bragg publication also anticipated the momentous problems facing crystallographers in pursuing their goals of unveiling the three-dimensional structures of their beloved crystals of ever-increasing complexity using the Fourier expansion of density in the crystals. There were three clear steps: (i) the measurement of complete and accurate intensities (amplitudes), (ii) the phasing of the structure factors derived from the intensities beyond the centric projections and (iii) the time-consuming work of making the appropriate calculations of increasing complexity.

Thus, a brief mention of the development of methods to expedite the calculation of Fourier transforms is appropriate. The computing aids and tools that were developed prior to the use of digital computers have been very concisely reviewed (McLachlan, 1983 ▸). Noteworthy are Beevers–Lipson strips, which were the main tool for many generations of crystallo­graphers (Fig. 4 ▸; Beevers & Lipson, 1934 ▸, 1936 ▸), and several variants that followed, as well as the use of punched cards and the associated sorting and manipulating machines. Also significant and instructive are the various optical devices (Taylor & Lipson, 1964 ▸; Bragg, 1939 ▸) that demonstrated the conceptual connection of X-ray diffraction to the ordinary light microscope and theories of optical imaging. The importance of digital computers in crystallography was patently demonstrated with the solution of the structures of the first proteins in the late 1950s and early 1960s, and has also been reviewed up to the mid-1980s (Sparks & Trueblood, 1983 ▸).

More importantly, in the context of Fourier methods in crystallography, is the development of fast and efficient algorithms to compute the Fourier transforms (both forwards and backwards and Patterson functions) necessary to solve and refine structures of ever larger complexity and size. Even the straight calculation of the electron-density function at high resolution, with a typical sampling of one-third of the resolution by means of the discrete Fourier transform (DFT), can be a daunting proposition, since the calculation scales as *N* ^2^ (where *N* is the number of electron-density map grid points). The introduction of fast Fourier transform (FFT) algorithms (Cooley & Tukey, 1965 ▸) scaled the calculations to the order of *N*log_10_ *N* and had a tremendous impact in crystallography after its implementation by Lynn Ten Eyck (Ten Eyck, 1973 ▸, 1977 ▸). This translates to an improvement of over two orders of magnitude for *N* = 1024 (*N* = 2^10^; Jacobson, 1983 ▸). Even with the limited random access memory resources of the time, it allowed structure solution by multiple isomorphous replacement and noncrystallographic symmetry averaging of the first plant viruses Tomato bushy stunt virus (Harrison *et al.*, 1978 ▸; Bricogne, 1976 ▸) and Southern bean mosaic virus (Abad-Zapatero *et al.*, 1980 ▸), with unit cells in the range 330–450 Å and data sets of the order of 300 000 reflections at a resolution of 2.8 Å. These methods, expanded to include phase extension and refinement by noncrystallographic symmetry, yielded the solution of the first animal virus (Human rhinovirus 14) a few years later (Rossmann *et al.*, 1985 ▸).

The introduction of FFT algorithms transformed the Fourier transform into a fundamental and ubiquitous tool not only in crystallography but also in the analysis of linear systems, optics, image reconstruction and analysis, including all variations of 3D tomography, analysis of random processes, probability and random variables, quantum physics and the solution of partial differential equations, among other fields in science and engineering. It is a tool common to many scientific endeavors and disciplines (Brigham, 1974 ▸).

Nearly two centuries after the mathematical and physical insights of J.-B. J. Fourier into the phenomena of heat transfer, I would like to pay homage to Fourier by discussing the developments of his work for the modern description of irreversible phenomena, the concept of time and the flow of time as related to entropy and non-equilibrium thermodynamics, all in the context of the aforementioned book by Professor Canales (Canales, 2015 ▸) and the work of the Nobel laurate Professor Ilya Romanovich Prigogine (1917–2003).

## 5. The Bergson–Einstein encounter and the debate over the concept of time   

Although it had multiple practical applications, the relevance and importance of Fourier’s equation for the transmission of heat in solids was not part of the mainstream of theoretical physics even a century after its publication. After World War I, the physics agenda was dominated by the theory of general relativity. Two British expeditions, one off the west coast of Africa and the other in northern Brazil, returned with astronomical observations confirming the predictions of the theory. Arthur Stanley Eddington (1882–1944; later Sir) returned from the 1919 Brazil eclipse expedition carrying with him photographic plates showing the bending of light by the gravitational field of the massive Sun. The meeting of the Royal Society in London where the Astronomer Royal for England announced the vindication of Einstein’s predictions crowned the physicist as the man who had supplanted Newton’s vision of the universe and done away with universal time. Time was merged with space, and only the space–time continuum was a valid description of our external world.

In this context, the Bergson–Einstein debate of 1922 ended with the implicit vindication of Einstein, given the weight of the recent experimental verification of the general theory of relativity. The conception of time as *durée* (duration), as expounded by the French philosopher, did not exist. Only personal time, ‘psychological time’ as stated by Einstein, existed. However, Sir Arthur S. Eddington, who wrote about his fortune to be present when the Astronomer Royal announced the results of the eclipse expeditions and was an earlier enthusiastic supporter of relativity, later raised significant objections to Einstein’s worldview and his conception of time as connected with the simultaneity of light signals. His scientific stand respected the experimental verification of relativity, but did not support its philosophical implications; he coined the term ‘the arrow of time’ (Eddington, 1927 ▸), connecting it to the one-directional increase in entropy.

From the viewpoint of the mathematical description of physical phenomena, as a legacy of Newtonian mechanics and dynamics, in statistical mechanics (Boltzmann), electromagnetism (Maxwell’s field equations), relativity, analytical and Hamiltonian formulations of dynamics and quantum mechanics all of the equations contain the variable of time (as *t* or d*t*) as a square term. Therefore, they are invariant to the reversal of time (changing *t* to −*t*). This can be illustrated using the equation for the propagation of a wave (D’Alembert equation derived from Maxwell’s field equations) *in vacuo* (only one spatial dimension is presented for simplicity; *c* is the velocity of propagation and *u* is the amplitude of the wave),
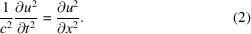



In contrast, the mathematical treatment of the propagation and transmission of heat demonstrated by Fourier in 1822 showed for the first time that the phenomenon depends on the first-order derivative of time on one side of the equation and the second derivative of the spatial coordinates on the other. These physical phenomena will have a ‘time direction’. This is certainly discernable to the casual observer, since the equation has the same mathematical dependence as the well known diffusion equation if one replaces the temperature *T* in Fourier’s equation by the concentration *C* in the more familiar Fick’s law of diffusion. These physico-chemical phenomena are irreversible and are mathematically described by non­equilibrium thermodynamics (*k* is the coefficient of heat conductivity of the material; Katchalsky & Curran, 1965 ▸),
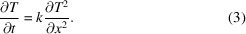



## 6. Mathematical description of irreversible phenomena and its implications   

The 20th century Belgian physical chemist Ilya Romanovich Prigogine, whose seminars I was fortunate enough to attend during my graduate student days at the University of Texas at Austin, had this conflict in mind from his early student days. Reviewing his nascent interest in the notion of time, he wrote

It is now more than fifty years since I published my first paper on nonequilibrium thermodynamics, in which I pointed out the constructive role of irreversibility. To my knowledge, this was also the first paper that dealt with self-organization as associated with distance from equilibrium. After so many years, I often wonder why I was fascinated with the problem of time, and why it took so very long to establish its relationship with dynamics.

(Prigogine, 1996 ▸).

Given his strong interest in the issues of time and non­equilibrium thermodynamics, Prigogine joined the existing school in Brussels founded by Théophile Ernest De Donder (1870–1957). Over the years, the work of Professor Prigogine and his associates at the University of Brussels and at the University of Texas at Austin has been published extensively in books, scientific articles and publications for wider audiences, with the latter publications discussing the philosophical implications of their technical findings. In the midst of his intense work on nonequilibrium thermodynamics and its implications for the concept of time in nonequilibrium processes, Professor Prigogine reviewed for *Nature* a series of essays on Bergson, including the translated transcripts of the Bergson–Einstein meeting in Paris, and wrote

Einstein gave a presentation of his theory of special relativity, and Bergson expressed some doubts about it. It is true that Bergson had not understood Einstein. But it is also true that Einstein had not understood Bergson. Bergson was fascinated by the role of creativity, of novelty in the history of the universe. But Einstein did not want any directed time. He repeated often that time, more precisely the arrow of time, is an ‘illusion’. So, these ideologies seem to be irreconcilable.

(Canales, 2015 ▸).

Prigogine’s scientific contributions, as well as those of his collaborators and colleagues, have attempted to reconcile some of Bergson’s insights with the laws of physics for over half a decade. I will present a brief summary and my own perspectives and future avenues, including some speculations on what could follow.

Even though Fourier’s memoir had been presented a century earlier, descriptions of the physical phenomena to which Fourier’s equation was applicable were not considered to be part of the Bergson–Einstein debate. Apparently, there was not any solid physical description of time that could be related to the ‘time duration’ as required to explain the irreversible processes that occur all around us in nature. Processes such as diffusion, heat transport, ‘dissipative structures’, evolution through fluctuations and related phenomena were beyond the scope of rigorous mathematical analysis and were therefore distrusted by theoretical physicists.

After the work of Prigogine and his colleagues at the University of Texas at Austin and in Belgium, as well as other scientists currently extending and exploring these ideas, now there is such a mathematically sound description of these processes. For processes that include the thermodynamic description of living systems (humans included), the distinction between ‘physical time’, understood as ‘physico-chemical time’, and ‘duration’ (‘*durée*’) does not exist. There could also be a physiological and personal dimension of time, but for this we still do not have a detailed physico-chemical explanation and description. Nonetheless, this could change in the second half of the 21st century as the study of the molecular, biochemical and neurological basis of the brain circuitry continues.

Einstein was defending what I would call ‘inertial or mechanical’ or better ‘dynamic’ time, related to motion and the transmission of signals and limited by the constant velocity of light, which is a key invariant in his vision of the universe. Beyond this, Einstein could not conceive of any other time except for psychological or personal time. After the work of Prigogine and venturing into the future, I would suggest that it is possible to conceptually distinguish three types of time.

(i) Inertial or dynamic time. This is tied to inertial systems in motion and is related completely to Einstein’s notion of time. This is the time of mechanical and dynamic phenomena in both classical and quantum mechanics; it is reversible and appears in equations as *t* ^2^ or d*t* ^2^ in the denominator of mechanical equations. The variable *t* can be changed to −*t* and nothing changes. Enough has been written about this and more so related to the Bergson–Einstein debate.
(ii) Physico-chemical time. This is connected to physico-chemical processes in which matter/energy are consumed and dissipated. It does relate to the concept of *durée* or duration defended by Bergson. The simplest and most striking example of this is the ‘burning of a candle’, with obvious reference to the life spans of organisms, human beings included. This time is directly related to irreversible processes in which heat is dissipated and implies a sense of direction, *i.e.* an arrow or flow of time. This is the gap that the work of Prigogine has filled with his rigorous, mathematically sound, concepts of ‘entropy production’ and ‘microscopic irreversibility’ and his distinctions as to the behavior of systems: ‘near equilibrium’ *versus* ‘far from equilibrium’. This distinction is important to understand the richness of the phenomena that can occur in the latter, and its implications for microscopic and macroscopic evolution: order through fluctuations, including macroscopic and biological evolution. This could be expanded to ‘biological time’ or ‘geological time’ as a subtype of physico-chemical time or as a category on its own.
(iii) Psychological time. This is perceived by humans and possibly also certain animals, and includes individual time perception and the ‘relativity’ of time spans among humans and among human situations. It is quite conceivable, in my view, that by the end of the current century, following advances in the study of the circuitry and the biochemical and molecular mechanisms of the brain, we may explain how we humans conceive and experience time, and the individual variations that each of us experiences. Pressing questions to answer could be: are there neural correlates for time? Is the perception of human time related to our own memories? Is it related to our own individuality as human beings? Is it related to consciousness? I could speculate that certain animal species could serve as animal models, in the same way that we have used ‘animal models’ to study psychological responses to other phenomena such as audition, music perception, language acquisition and spatial skills; there is so much that we do not yet know.

## 7. Continuing the dialog with nature   

In her book about the Bergson–Einstein debate, Professor Canales discusses in a final section (Postface) one of the books presenting the implications of Prigogine’s work for a wider audience: *Order Out of Chaos, Man’s New Dialog with Nature*. She quotes the authors

Physics, today, no longer denies time. It recognizes the irreversible time of evolutions toward equilibrium, the rhythmic time of structures whose pulse is nourished by the world they are part of, the bifurcating time of evolutions generated by instability and amplifications of fluctuations, and even microscopic time, which manifests the indetermination of microscopic physical evolutions.

(Prigogine & Stengers, 1984 ▸) and she asks: has science vindicated Bergson?

She admits that a few thinkers were enthusiastic about the ‘new alliance’, but the majority did not embrace the new vision positively. What Professor Canales describes as ‘science wars’ ensued in the 1990s, confronting physicists and humanists with arguments that could be traced back to the Bergson–Einstein debate. She argues that the controversy rages on and is still feeding the gap between science and the humanities. In my view, this interpretation is only partially correct and incomplete.

Prigogine’s Nobel Prize lecture in 1977 acknowledges that the new avenues opened by the study of irreversible phenomena have resulted in a rapprochement of the different notions of time in theoretical physics and in other aspects of the world we live in:

These questions will probably be clarified in the coming years. But already now the development of the theory permits us to distinguish various levels of time: time as associated with classical or quantum dynamics, time associated with irreversibility through a Lyapounov function and time associated with ‘history’ through bifurcations. I believe that this diversification of the concept of time permits a better integration of theoretical physics and chemistry with disciplines dealing with other aspects of nature.

(Prigogine, 1977 ▸).

Expanding briefly on Prigogine’s quote, we can distinguish three levels of time. Firstly, Einstein’s time and the time of classical and quantum dynamics, where the variable time (*t*) appears as a second derivative and therefore processes can run reversibly for *t* and −*t*. Secondly, ‘Bergsonian’ time, individual time, as in the ‘burning of a candle’, diffusion processes, heat transport and human life, *i.e.* duration. Time is only present in the equations as the first derivative; thus, the processes are not reversible. This is time as in irreversible processes near equilibrium. Finally, ‘creative evolution’ time as in the evolution of processes far from equilibrium subject to ‘bifurcation points’ (unpredictability), possibly with extension, among others, to the evolution of macroscopic processes and conceivably to ‘macroscopic biological evolution’, *e.g.* Darwinian evolution.

Considering the importance that time has had through the centuries in philosophy and human culture, I would dare to extend the last sentence of Prigogine’s quotation to read

I believe that this diversification of the concept of time permits a better integration of theoretical physics and chemistry with disciplines dealing with other aspects of nature and *human culture, including philosophy*.

(italics added for my slight revision and emphasis).

Unfortunately, from this point on Canales’s text emphasizes the controversy of the so-called ‘science wars’ between physicists and humanists, without emphasizing and explaining, bringing down to the level of the reader, the findings of Prigogine and his colleagues and their implications to clarify, and put into scientific terms, some of Bergson’s insights.

There is an extensive review of the viewpoints of the different philosophers and schools that followed after the Bergson–Einstein debate. Regrettably, if Bergson did not understand Einstein, the current philosophers have not digested or fully understood the enormous influence and ‘explanatory power’ of Prigogine’s studies to illuminate the concepts of ‘time’ that were alien to Einstein. These concepts are now familiar to scientists studying physico-chemical and biological phenomena at the molecular and cellular levels (Babloyantz, 1986 ▸), and our macromolecular and structural insights will be essential elements in fully understanding them in the future. I will conclude with a quotation from Professor Prigogine:

I have always considered science to be a dialog with nature. As in a real dialog, the answers are often unexpected — and sometimes astonishing.

(Prigogine, 1996 ▸, p. 57).

In summary, the contributions of J.-B. J. Fourier to crystallography and basic and applied mathematics nearly two centuries ago were instrumental in understanding important physical problems of his time such as the transmission of heat in solid objects. In addition, he opened the field of the mathematical treatment of irreversible phenomena related to the future concepts of thermodynamics, entropy and the flow of time. In the 20th century, the ensuing analyses and study of irreversible phenomena, as developed by the work of Prigogine and his school, has opened the doors to other ‘physical’ conceptions of time well beyond Einstein’s limited view that made him collide with Bergson’s philosophical insights. We should be cognisant and proud of the fact that J.-B. Joseph Fourier, who provided the essential conceptual and mathematical methods for crystallography among many other scientific achievements, also opened the first window to reveal the connection between irreversible processes and the processes related to our *durée* as human beings.
